# Association of the ACTN3 R557X polymorphism with glucose tolerance and gene expression of sarcomeric proteins in human skeletal muscle

**DOI:** 10.14814/phy2.12314

**Published:** 2015-03-16

**Authors:** Isabelle Riedl, Megan E Osler, Boubacar Benziane, Alexander V Chibalin, Juleen R Zierath

**Affiliations:** 1 Department of Molecular Medicine and Surgery, Section for Integrative Physiology, Karolinska InstitutetStockholm, Sweden; 2 Department of Physiology and Pharmacology, Section for Integrative Physiology, Karolinska InstitutetStockholm, Sweden

**Keywords:** Glucose metabolism, sarcomere, type 2 diabetes

## Abstract

A common polymorphism (R577X) in the *α*-actinin (ACTN) 3 gene, which leads to complete deficiency of a functional protein in skeletal muscle, could directly influence metabolism in the context of health and disease. Therefore, we tested the hypothesis that states of glucose tolerance are associated with the ACTN3 R577X genotype. We analyzed the prevalence of the ACTN3 R577X polymorphism in people with normal glucose tolerance (NGT) and type 2 diabetes (T2D) and measured muscle-specific *α*-actinin 2 and 3 mRNA and protein abundance in skeletal muscle biopsies. Furthermore, we investigated the protein abundance of the myosin heavy chain isoforms and the components of the mitochondrial electron transport chain in skeletal muscle from people with NGT or T2D. mRNA of selected sarcomeric z-disk proteins was also assessed. Although the prevalence of the ACTN3 577XX genotype was higher in T2D patients, genotype distribution was unrelated to metabolic control or obesity. ACTN2 and ACTN3 mRNA expression and protein abundance was unchanged between NGT and T2D participants. Protein abundance of mitochondrial complexes II and IV was related to genotype and glucose tolerance status. Gene expression of sarcomeric z-disk proteins was increased in skeletal muscle from NGT participants with the ACTN3 577XX genotype. While genetic variation in ACTN3 does not influence metabolic control, genotype does appear to influence gene expression of other sarcomeric proteins, which could contribute to the functional properties of skeletal muscle and the fatigue-resistant phenotype associated with the R577X polymorphism.

## Introduction

Skeletal muscle has important function in structure and locomotion, as well as in whole-body metabolism. Type 2 diabetes (T2D) is a metabolic disease characterized by insulin resistance and impaired glucose metabolism in skeletal muscle (DeFronzo et al. 1981). Individual susceptibility to T2D and related metabolic diseases varies widely, and is modulated by genetic and environmental factors. Numerous studies of candidate gene, genome-wide association, and linkage have identified gene variants or genetic backgrounds associated with T2D and glucose homeostasis (Bonnefond et al. 2010; Dimas et al. 2014).

A single-nucleotide polymorphism (SNP) has been identified in the muscle-specific *α*-actinin 3 (*ACTN3*) gene (R577X, rs1815739), which results in a premature stop codon (North et al. 1999). *α*-actinin 3 protein deficiency is prevalent in 16–18% of the Caucasian population (North et al. 1999). This SNP is associated with a shift toward an oxidative metabolism through altered calcineurin signaling (Seto et al. 2013), sarcomere composition (Seto et al. 2011), and glycogen metabolism (Quinlan et al. 2010; Norman et al. 2014). Despite a strong association between ACTN3 R577X genotypes and performance in highly trained athletes, the relationship between this SNP and T2D is unknown.

Members of the *α*-actinin (ACTN1-4) family of proteins are highly conserved actin-binding proteins that influence sarcomere integrity by cross-linking actin molecules filaments (Blanchard et al. 1989). In human skeletal muscle, the ACTN2 isoform is expressed in glycolytic and oxidative fiber types, while ACTN3 is restricted to glycolytic fibers (North and Beggs 1996). Initially, the *α*-actinins were strictly considered to be structural proteins, but the discovery of the ACTN3 R577X polymorphism highlights a metabolic role for *α*-actinin 3 in skeletal muscle (Berman and North 2010). *α*-actinin 3 deficiency has been investigated in the context of McArdle's disease (Lucia et al. 2007), spinal cord injury (Broos et al. 2012), aging (Zempo et al. 2011), and muscle immobilization (Garton et al. 2014), but not in T2D or obesity.

In elite athletes, a higher proportion of individuals carrying the 577XX genotype excel in low-intensity aerobic activities (Yang et al. 2003; Eynon et al. 2009; Shang et al. 2010), while a lower frequency of the X allele is associated with elite performance in high-intensity anaerobic sports, such as sprinting (Druzhevskaya et al. 2008; Papadimitriou et al. 2008; Roth et al. 2008). In mice, genetic deletion of ACTN3 favors oxidative metabolism (MacArthur et al. 2007) and increased abundance of several sarcomeric proteins interacting with the *α*-actinins (Seto et al. 2011). Moreover, in humans and mice, the absence of *α*-actinin 3 alters the interaction between *α*-actinin 2 and the sarcomere, consequently modulating calcineurin signaling (Seto et al. 2013). Thus, we hypothesized the ACTN3 R577X genotype may impact glucose metabolism, as well as abundance and expression of mitochondrial and sarcomeric proteins in skeletal muscle.

Here, we characterized the prevalence of the ACTN3 R577X polymorphism in people with normal glucose tolerance (NGT) or T2D and assessed the relationship of genotype to anthropometric and metabolic characteristics. We also determined the association between the ACTN3 R577X genotypes and skeletal muscle myosin heavy chain isoforms, mitochondrial complex enzymes, and the contractile network. We report a higher prevalence of the 577XX genotype among T2D patients, although measures of metabolic control were unaffected by this genotype. We also provide evidence that the ACTN3 R577X polymorphism modulates mRNA of sarcomeric proteins, thereby affecting structural and functional integrity of human skeletal muscle. Collectively, these results provide insight into the impact of the ACTN3 R577X polymorphism on skeletal muscle physiology.

## Methods

### Participants

A total of 177 male and female volunteers were included in the study. This cohort represents a subgroup of participants studied earlier by our group (Fritz et al. 2011, 2013). A portion of the clinical data presented in Table1 has also been reported earlier (Fritz et al. 2011, 2013). Participants were classified as NGT or T2D following an oral glucose tolerance test (OGTT) performed as described (Fritz et al. 2011). The inclusion criteria for all groups were an age range of 45–69 years old and a BMI > 25 kg/m^2^. T2D patients had HbA_1c_ levels ranging from 7.4% to 9.8%. The exclusion criteria were physical impairments, symptomatic angina pectoris, atrial fibrillation measured by ECG, systolic or diastolic blood pressure >160 and >100 mmHg, respectively, or insulin treatment. Upon enrollment in the study participants underwent a complete medical examination and their anthropometric and metabolic characteristics were measured. Following an overnight fast, participants underwent an OGTT (Fritz et al. 2011). Cardiorespiratory fitness was assessed by measuring oxygen uptake using a ramp test on a mechanically braked ergometer as described previously (Fritz et al. 2013). Participants provided written informed informal consent and all protocols were approved by Karolinska Institutet ethics committee.

**Table 1:** Anthropometric and metabolic traits of the study participants

	NGT	T2D
*n*	128	49
Sex (M/F)	47/81	31/18
Age (y)	59 ± 1	61 ± 1
Height (cm)	170 ± 1	172 ± 1
Weight (kg)	84.7 ± 1.0	92.8 ± 2.1*
Waist circumference (cm)	98.2 ± 0.9	104.6 ± 1.5*
BMI (kg/m^2^)	29.5 ± 0.3	31.4 ± 0.6*
Fasting glucose (mmol/L)	5.5 ± 0.0	7.9 ± 0.2*
2-h glucose (mmol/L)	7.2 ± 0.1	15.4 ± 0.6*
Insulin (pmol/L)	57.5 ± 3.0	69.5 ± 6.2
HbA1c (%)	4.7 ± 0.0	6.1 ± 0.1*
HOMA-IR	2.0 ± 0.1	3.5 ± 0.3*
Workload (W)	157 ± 4	158 ± 5.5
Oxygen Uptake (mL × min^−1^ × kg^−1^)	24.0 ± 0.6	22.9 ± 1.0

Data are mean ± SEM. HOMA-IR, homeostasis model assessment – estimated insulin resistance; W, watts. Statistical comparison between NGT versus T2D participants was determined using Student's t-test or Mann-Whitney *U*-test. *P <* *0.05 for NGT versus T2D.

### Skeletal muscle biopsies

Skeletal muscle biopsies were obtained from a subset of male and female participants with the three different ACTN3 R577X genotypes. Biopsies (20–100 mg) were obtained from the *vastus lateralis* portion of the *quadriceps femoris* muscle using a conchotome tongue (Dietrichson et al. 1987) after overnight fast and transferred to liquid nitrogen.

### DNA extraction and genotyping

Venous blood was collected from the participants after an overnight fast and stored in Vacutainer® tubes containing EDTA and stored at −80°C for further analysis. Genomic DNA (gDNA) was extracted from 200 *μ*L of whole blood using a commercial kit (Qiagen, Hilden, Germany), according to the manufacturer's instructions. Using 10 *μ*g of input gDNA, genotyping for ACTN3 R577X SNP was performed by PCR amplification (10 min at 95°C followed by 40 cycles of 15 sec at 95°C for 1 min at 60°C) followed by allelic discrimination assay with fluorogenic probes (1 min at 60°C) (TaqMan® SNP Genotyping Assays, ID C_590093_1; Applied Biosystems, Foster City, CA) using a thermal cycler (ABI 7000, Applied Biosystems).

### Protein extraction and protein abundance analysis

Skeletal muscle biopsies were freeze-dried overnight. Muscle fibers were dissected out and cleaned from blood and connective tissue under binocular microscope. Samples were homogenized in ice-cold homogenization buffer [20 mmol/L Tris, pH 7.8, 137 mmol/L NaCl, 2.7 mmol/L KCl, 1 mmol/L MgCl_2_, 0.5 mmol/L Na_3_Vo_4_, 1% Triton X-100, 10% glycerol, 10 mmol/L NaF, 0.2 mmol/L phenylmethylsulfonyl fluoride, 1 mmol/L EDTA, 5 mmol/L Na_4_P_2_O_7_, 1% (v/v) Protease Inhibitor Cocktail (Calbiochem, Darmstadt, Germany)] using a motor-driven pestle. Lysates were rotated for 1 h at +4°C and subjected to centrifugation at 12,000 g for 10 min at +4°C. Supernatants were collected and protein concentrations were determined using a bicinchoninic acid protein assay kit (Pierce, Rockford, IL). Protein lysates were dissolved in 4× Laemmli buffer, separated by SDS-PAGE using Bis-Tris Criterion XT Pre-cast gels (Bio-Rad, Sundbyberg, Sweden), blocked in 7.5% milk for 1 h at room temperature, and probed overnight at +4°C with antibodies against *α*-actinin 2 and *α*-actinin 3 (1:250,000 and 1:1000, respectively, kind gifts from K. North). An antibody cocktail that recognizes the NDUFB8 (complex I), SDHB (complex II), UQCR2 (complex III), COX II (complex IV) and ATP5A (complex V) enzymes of the electron transport chain was acquired from Abcam (#ab110411, Nordic Biosite, Sweden). Antibodies recognizing MyHC-1, -2A, -2B (#BA-D5, SC-71, and BF-F3, respectively) were purchased from the Developmental Studies Hybridoma Bank (University of Iowa, Iowa). The antibody for GAPDH was from Santa Cruz Biotechnologies (#sc-25778, Solna, Sweden). Membranes were washed and incubated in the appropriate secondary antibody (Bio-Rad) for 1 h at room temperature and subsequently subjected to enhanced chemiluminescence detection reagents (GE healthcare, Waukesha, WI) to visualize the proteins. All quantifications were performed using a positive control to control for intergel variability.

### RNA extraction and gene expression measurement

Total RNA was extracted from 25 to 30 mg of skeletal muscle using Trizol reagent (Invitrogen, Carlsbad, CA) and 1–1.5 *μ*g of RNA was used to synthesize cDNA (SuperScript First Strand Synthesis System, Invitrogen). TaqMan® gene expression assays were used (*ACTA1* Hs00559403_m1, ACTN3 Hs01100111_g1, *ACTN2* Hs01552477_m1, *CAPZA1* Hs04187789_g1, *CAPZA2* Hs00255135_m1, *CAPZB* Hs01120796_m1, *LDB3* Hs00951222_m1, *MYOT* Hs00199016_m1, *MYOP* Hs00261515_m1, *MYOZ1* Hs00222007_m1, *MYOZ2* Hs00213216_m1, *MYOZ3* Hs00911018_s1, *NEB* Hs00189880_m1, *PDLIM3* Hs01062534_m1, *PDLIM6* Hs00253222_m1, *TTN* Hs00399225_m1) to measure gene expression by quantitative real-time PCR (StepOnePlus™ Real-Time PCR System; Applied Biosystems). Reactions were performed in duplicate in a 96-well format. Relative gene expression was calculated using the comparative C_T_ method and normalized to a selected housekeeping gene (*β*2-microglobulin) for internal control.

### Statistical analysis

Differences in anthropometric measurements and clinical parameters between NGT and T2D (Table1) were assessed using Student's *t*-test or Mann–Whitney *U*-test when the data were not normally distributed. For genotype distribution, Hardy–Weinberg equilibrium was tested using the Chi-squared test. Anthropometric measurements and clinical parameters in relation to genotype (Table3) were analyzed with a Kruskal–Wallis test to evaluate differences between groups. Gene expression and Western blot quantifications analysis from Figs.3 were performed using two-way ANOVA to examine both effects of genotype and glucose tolerance status. Pairwise post hoc comparisons were determined using Bonferroni correction to control for type 1 error. Data are presented as mean ± SEM. Statistical analyses were performed using Graphpad or SPSS.

**Figure 1:**
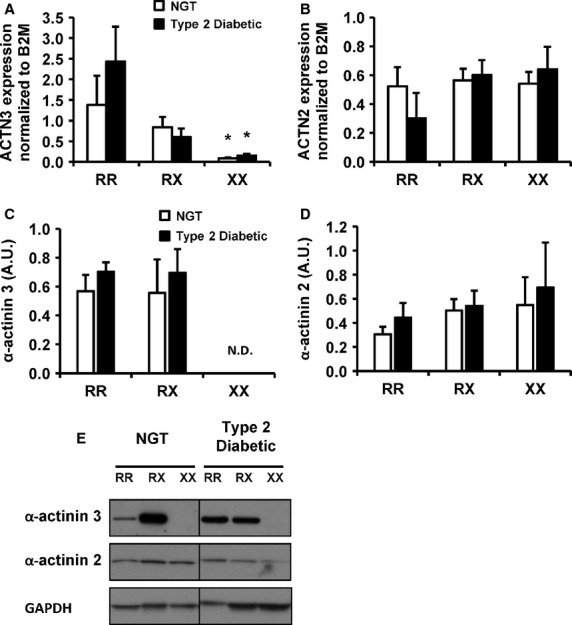
mRNA expression and protein abundance of ACTN2 and ACTN3. Gene expression of ACTN isoforms 2 and 3 was measured in skeletal muscle from individuals with NGT or T2D. (A) ACTN3 mRNA expression. (B) ACTN2 mRNA expression. *N *=* *4–8 per genotype per condition. Results are normalized to *β*2-microglobulin. Level of *α*-actinin 2 and 3 was measured in protein lysates prepared from skeletal muscle from individuals with NGT or T2D. (C) Quantification of *α*-actinin 3 protein. (D) Quantification of *α*-actinin 2 protein. (E) Representative Western blot for protein abundance of *α*-actinin 2 and 3, and GAPDH. *N *=* *5–6 per genotype per condition. N.D.: nondetectable. Results are mean ± SEM. *P ≤* *0.01 for 577RR versus 577XX in each cohort.

**Figure 2:**
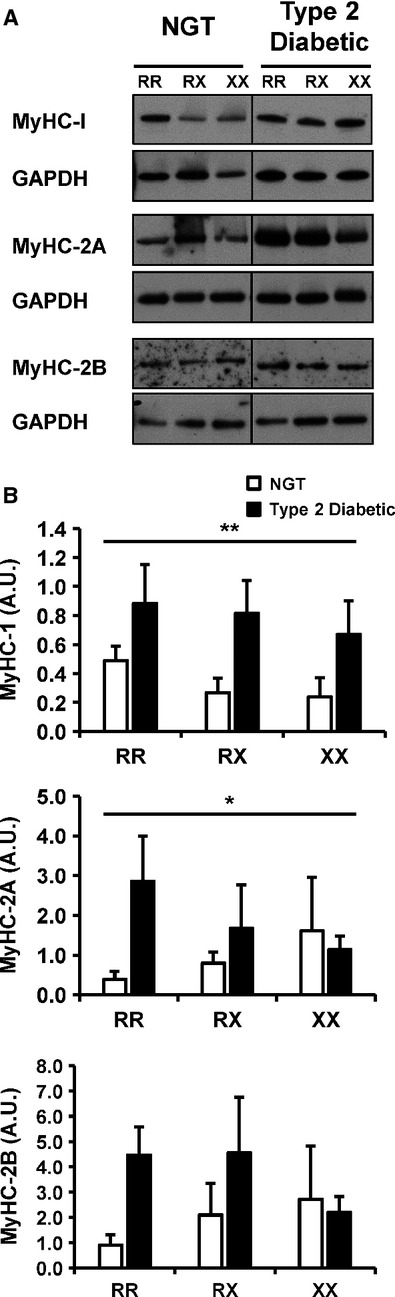
Protein abundance of the human myosin heavy chain isoforms. Abundance of myosin heavy chain isoforms was measured in skeletal muscle from individuals with NGT or T2D. (A) Representative Western blot of protein abundance of myosin heavy chain 1, 2A, 2B, and GAPDH. (B) Quantification of myosin heavy chain 1, 2A, and 2B in skeletal muscle from individuals with the ACTN3 577RR, RX, or XX genotype. *N *=* *4–6 per genotype per condition. Results are mean ± SEM. *P ≤* *0.05; **P ≤* *0.01 for glucose tolerance effect.

**Figure 3:**
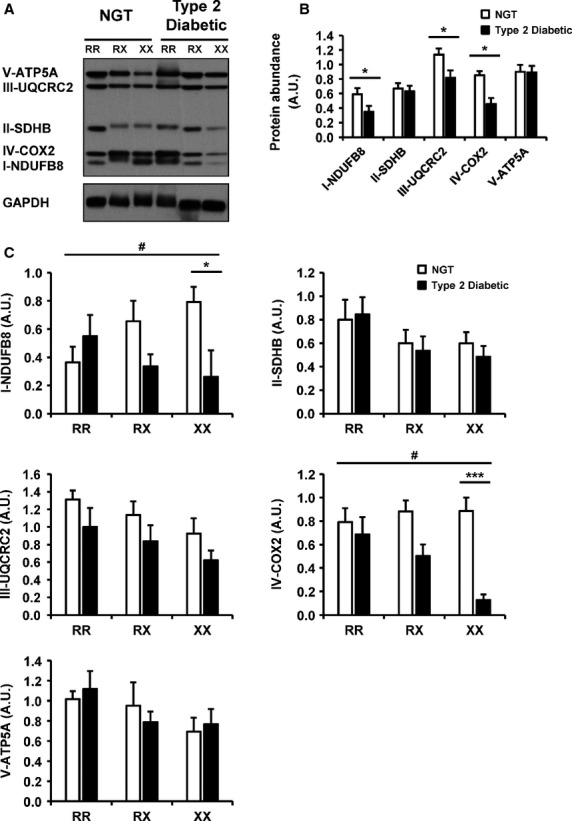
Protein abundance of mitochondrial complex enzymes. Abundance of individual mitochondrial electron chain transport components was measured in skeletal muscle from individuals with NGT or T2D. (A) Representative Western blot for protein abundance of five mitochondrial enzymes in skeletal muscle from individuals with the ACTN3 577RR, RX, or XX genotype. (B) Quantification of mitochondrial enzymes in skeletal muscle from individuals with NGT or T2D, regardless of genotype. *N *=* *15–18 per condition. (C) Quantification of mitochondrial enzymes in skeletal muscle from individuals with NGT or T2D, according to genotype. *N *=* *5–6 per genotype per condition. Results are mean ± SEM. *P ≤* *0.05; ***P ≤* *0.001 for 577XX NGT versus 577XX T2D; ^#^*P *≤* *0.05 for an interaction between genotype and glucose tolerance status.

## Results

### ACTN3 genotype distribution

To explore whether the ACTN3 R577X genotype impacts metabolic status, a cohort of NGT and T2D individuals (*n *=* *177) (Fritz et al. 2011, 2013) was genotyped for the polymorphism. Body weight, waist circumference, body mass index (BMI), fasting blood glucose, 2 h blood glucose, glycosylated hemoglobin (HbA_1c_) levels, and homeostasis model assessment – estimated insulin resistance (HOMA-IR) levels differed between the NGT and T2D participants (*P *<* *0.05) (Table1). The genotype distribution of the complete cohort studied met the Hardy–Weinberg equilibrium, with the R and X allele reaching frequencies of 0.6 and 0.4, respectively (Table2). A higher frequency of the 577XX genotype was detected in individuals with T2D versus NGT (*P *=* *0.039) (Table2). However, across both NGT and T2D, anthropometric and metabolic parameters were unrelated to the ACTN3 genotype (Table3).

**Table 2:** ACTN3 genotype distribution and allele frequencies

	*n*	RR (%)	RX (%)	XX (%)	*P* value
NGT	128	51 (40)	61 (48)	16 (12)	*P =* *0.039
T2D	49	11 (22)	26 (53)	12 (24)	
Total	177	62 (35)	87 (49)	28 (16)	

Statistical comparison between groups was performed using a Chi-squared test. *P indicates genotype distribution difference between participants with NGT versus T2D.

**Table 3:** Association between ACTN3 genotype with anthropometric and metabolic traits

Genotype	Normal glucose tolerant			Type 2 diabetic		
	RR	RX	XX	RR	RX	XX
*n*	51	61	16	11	26	12
Sex (M/F)	21/30	20/41	6/10	37/39	16/10	6/6
Age (y)	60 ± 1	60 ± 1	56 ± 2	58 ± 2	63 ± 1	60 ± 1
Weight (kg)	85.9 ± 1.9	84.0 ± 1.3	83.9 ± 3.2	94.4 ± 4.0	93.7 ± 3.1	89.2 ± 3.8
Waist (cm)	99.0 ± 1.5	98.3 ± 1.1	95.1 ± 2.7	103.8 ± 2.2	105.1 ± 2.4	104.1 ± 2.8
BMI (mg/kg^2^)	29.5 ± 0.4	29.5 ± 0.4	29.2 ± 1.0	30.5 ± 1.0	31.7 ± 0.9	31.8 ± 1.4
f-glucose (mmol/L)	5.4 ± 0.1	5.5 ± 0.1	5.6 ± 0.1	8.1 ± 0.7	7.8 ± 0.2	8.0 ± 0.5
2-h glucose (mmol/L)	7.2 ± 0.1	7.2 ± 0.1	7.1 ± 0.2	15.3 ± 1.4	14.6 ± 0.7	16.9 ± 1.1
f-insulin (pmol/L)	62.4 ± 5.2	56.8 ± 4.5	43.8 ± 4.4	80.8 ± 16.8	62.9 ± 7.6	72.9 ± 9.1
HbA1c (%)	4.7 ± 0.1	4.7 ± 0.1	4.6 ± 0.1	6.2 ± 0.4	6.0 ± 0.2	6.2 ± 0.2
HOMA-IR	2.18 ± 0.18	2.00 ± 0.15	1.58 ± 0.17	4.00 ± 0.79	3.09 ± 0.37	3.76 ± 0.54
Workload (W)	163 ± 6	150 ± 5	166 ± 11	171 ± 12	155 ± 8	155 ± 8
Oxygen Uptake	24.3 ± 0.9	23.3 ± 0.9	25.9 ± 1.7	24.5 ± 1.8	21.6 ± 1.5	24.4 ± 1.6

Data are mean ± SEM. HOMA-IR, homeostatic model assessment insulin resistance; W, watts. Oxygen uptake is reported as mL × min^−1^ × kg^−1^. Statistical comparison of genotype within groups was performed using the Kruskal–Wallis test.

### α-actinin 2 and α-actinin 3 expression and abundance

To determine whether *α*-actinin levels vary with glucose tolerance, mRNA and protein levels of *α*-actinin 2 and *α*-actinin 3 were measured in *vastus lateralis* muscle from individuals with NGT or T2D. ACTN3 mRNA expression (*P *<* *0.001 and <0.01, respectively, Fig.1A) and *α*-actinin 3 protein abundance (Fig.1C) was lower in skeletal muscle of NGT and T2D carriers of the 577XX genotype versus 577RR. Conversely, ACTN2 mRNA (Fig.1B) and *α*-actinin 2 protein abundance (Fig.1D) was unaltered. Representative immunoblots for *α*-actinin 3 and *α*-actinin 2 protein across genotypes for people with NGT or T2D are shown and compared against GAPDH (Fig.1E). *α*-actinin 2 and *α*-actinin three expression and abundance across the different genotypes did not differ between people with NGT or T2D.

### Protein abundance of myosin heavy chain isoforms and mitochondrial complex enzymes

Skeletal muscle ACTN3 expression is restricted to fast glycolytic type 2 fibers. Thus, we determined whether the ACTN3 R577X polymorphism is related to fiber-type distribution and elements of the mitochondrial electron transport chain complexes. Protein abundance of three myosin heavy chain isoforms (MyHC-I, -2A, -2B) was determined in skeletal muscle from individuals with NGT or T2D carrying the three different ACTN3 R577X genotypes (Fig.2A). There was no interaction between genotype and glucose tolerance, or genotype alone on the protein abundance of any of the investigated targets. However, there was an interaction between glucose tolerance status and MyHC-1 and MyHC-2a (*P *=* *0.01 and *P *=* *0.03, respectively, Fig.2B), with T2D patients displaying increased protein abundance compared to NGT individuals. Protein abundance of MyHC-2B was unaffected by ACTN3 genotype or metabolic status (Fig.2B). To determine whether ACTN3 genotype influences components of the mitochondrial electron transport chain, protein abundance of individual enzymes was measured in lysates prepared from skeletal muscle from individuals with NGT or T2D with different ACTN3 R577X genotypes. Protein abundance of components from complexes I, III, and IV was decreased in people with T2D compared with NGT (Fig.3A and B). Protein abundance of NDUFB8 (complex I) and COX2 (complex IV) showed an interaction between genotype and glucose tolerance status (*P *=* *0.04 and *P *=* *0.03, respectively, Fig.2C). Protein abundance of these complexes was increased across genotype in NGT individuals, while in T2D patients, an inverse relationship was observed. An interaction between glucose tolerance and UQCRC2 (complex III) protein abundance was also observed.

### mRNA expression of sarcomeric proteins in skeletal muscle

**Figure 4:**
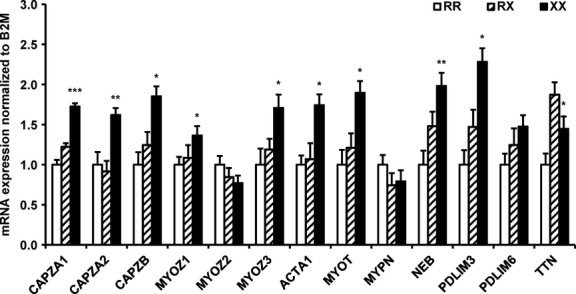
mRNA expression of sarcomeric proteins. mRNA expression of selected sarcomeric proteins was determined in skeletal muscle from NGT individuals with the ACTN3 577RR, RX or XX genotype. *N *=* *9–12 per genotype. Results are normalized to *β*2-microglobulin and presented as mean ± SEM.*P ≤* *0.05; **P ≤* *0.01; ***P ≤* *0.001 for 577RR versus 577XX.

*α*-actinin 3 is a structural protein that interacts with other sarcomeric proteins to maintain skeletal muscle z-line integrity. To determine the association between the ACTN3 R577X polymorphism and gene expression of *α*-actinin 3-binding partners in human skeletal muscle, mRNA expression of sarcomeric, and z-disk proteins was measured in *vastus lateralis* muscle (Fig.4). Gene expression of actin (ACTA1), ACTN2, capping protein isoforms (CAPZA1/2, CAPZB), myotilin (MYOT), myozenin/calsarcin isoforms (MYOZ1/2/3), myopalladin (MYPN), PDZ and LIM domain isoforms (PDLIM3/6), nebulin (NEB) and titin (TTN) was measured in individuals with NGT. mRNA expression of ACTA1, all capping protein isoforms, MYOZ1 and 3, MYOT, NEB, PDLIM3, and TTN was increased in skeletal muscle obtained from individuals carrying the R577X ACTN3 polymorphism.

## Discussion

The ACTN3 R577X polymorphism is associated with glycogen metabolism (Quinlan et al. 2010), calcineurin signaling (Seto et al. 2013), and oxidative metabolism (MacArthur et al. 2007, 2008). Thus, level of *α*-actinin 3 protein in skeletal muscle may play a role in metabolic diseases. Here, we tested the hypothesis that states of glucose tolerance are associated with the ACTN3 R577X genotype. As expected, ACTN3 mRNA expression and *α*-actinin 3 protein abundance mirrored the genotype distribution across the entire cohort. We report an increased prevalence of the 577XX genotype in people with T2D versus NGT. The ACTN3 R577X genotype was unrelated to metabolic control. In contrast to previous findings in women across the adult lifespan (Walsh et al. 2008), we did not note a relationship between genotype and BMI. Rather, our results support the notion that compensatory structural adaptations within the skeletal muscle sarcomere may account for the enhanced oxidative metabolism in individuals homozygous for the ACTN3 R577X genotype.

In humans carrying the ACTN3 R577X genotype and ACTN3 KO mice, *α*-actinin 2 protein is elevated (MacArthur et al. 2007; Seto et al. 2013), suggesting a compensatory role of this isoform. However, in our study, ACTN2 mRNA and *α*-actinin 2 protein levels were unaltered across the ACTN3 genotypes, consistent with another clinical investigation (Norman et al. 2009). Due to high sequence homology (>80%) of *α*-actinin 2 and *α*-actinin 3, a functional redundancy between these isoforms may mask a disease phenotype arising from an ACTN3 deficiency (Beggs et al. 1992). Thus, the ACTN3 R577X polymorphism does not appear to influence metabolic regulation in humans.

Since ACTN3 gene expression is restricted to fast glycolytic muscle fibers (North and Beggs 1996), we determined the relationship between the ACTN3 R577X polymorphism and skeletal muscle fiber type or the abundance of different mitochondrial complex proteins. Although the percentage of type IIB fibers is increased in healthy young individuals with the 577RR genotype (Vincent et al. 2007), the ACTN3 R577X polymorphism did not influence fiber-type distribution in our study, consistent with a recent report of young healthy men and women (Norman et al. 2014). Furthermore, skeletal muscle fiber-type distribution is unaltered between ACTN3 KO and wild-type mice, despite enhanced oxidative metabolism (MacArthur et al. 2008), thereby uncoupling the ACTN3-induced metabolic improvements from fiber-type changes. Protein abundance of MyHC-1 and MyHC-2A was increased in skeletal muscle from T2D patients. While the biological relevance of the increase in MyHC-1 is unclear, MyHC-2A protein abundance has previously been shown to be increased in skeletal muscle from T2D patients (Oberbach et al. 2006). Despite the increase in MyHC-2A protein abundance, level of *α*-actinin 2 and *α*-actinin 3 was unaltered in T2D patients. Protein abundance of mitochondrial transport chain enzymes NDUFB8 (complex I), UQCRC2 (complex III), and COX2 (complex IV) was reduced in skeletal muscle from T2D patients compared to NGT, consistent with a previous study (Oberbach et al. 2006). Interestingly, protein abundance of NDUFB8 and COX2 appear to be related to both ACTN3 R577X genotype and glucose tolerance status. Protein abundance of these oxidative enzymes increased with the number of X alleles in NGT individuals, whereas an inverse relationship was noted in T2D patients. Thus, the 577XX genotype is insufficient to protect against the decreased abundance of oxidative enzymes characteristic of T2D.

The ACTN3 R allele is associated with increased strength and power (Yang et al. 2003), but the mechanism by which *α*-actinin 3 modulates muscle performance from a structural perspective has remained elusive. Recent studies using ACTN3 KO mice link ACTN3 deficiency to altered skeletal muscle performance through calcineurin signaling pathways (Seto et al. 2013; Garton et al. 2014). *α*-actinin proteins function to stabilize and preserve the integrity of the sarcomere through interactions with other z-disk proteins (Papa et al. 1999; Pappas et al. 2008). Therefore, we investigated whether additional sarcomeric proteins found in the structural network of human skeletal muscle are influenced by the ACTN3 genotype. Here, we report that mRNA expression of sarcomeric and contractile proteins are increased in skeletal muscle of NGT carriers of the 577XX genotype, including ACTA1, CAPZA1 and 2, CAPZB, MYOZ1 and 3, MYOT, NEB, PDLIM3, and TTN. These results are further supported by findings from the ACTN3 KO mouse, in which mRNA expression of MYOT, PDLIM3, and PDLIM6 is increased in skeletal muscle (Seto et al. 2011, 2013). MYOZ1 and MYOZ3, two of the upregulated genes identified in carriers of the ACTN3 R577X polymorphism, are skeletal muscle-specific calcineurin-interacting proteins that also bind and colocalize to *α*-actinins (Frey et al. 2000).

Calsarcin-2, an inhibitor of calcineurin activation and encoded by the MYOZ1 gene, preferentially binds *α*-actinin 2 in ACTN3 KO mice, thereby reprogramming the skeletal muscle fiber profile to favor an endurance phenotype (Seto et al. 2013). Mice deficient for MYOZ1 display an increased endurance exercise capacity and a shift in skeletal muscle fiber properties, but no change in ACTN3 mRNA expression (Frey et al. 2008). Thus, our findings support earlier evidence implicating calcineurin as a key modulator of skeletal muscle reprogramming associated with the ACTN3 polymorphism (Seto et al. 2013). mRNA expression of CAPZA1 and 2, CAPZB, and nebulin was also elevated in skeletal muscle from carriers of 577XX genotype. Capping proteins and nebulin interact to stabilize the length of the actin filament (Pappas et al. 2008), enhancing skeletal muscle integrity, which could improve the response to repeated chronic contraction and contribute to a fatigue-tolerant phenotype. Lack of ACTN3 results in a coordinate increase in gene expression of sarcomeric proteins, which begets the phenotype observed in both the ACTN3 KO mouse and elite endurance athletes carrying the 577XX genotype.

Some of the limitations of this study include a focus on the relationship between the 577XX genotype and mRNA, rather than abundance of sarcomeric proteins. We attempted to assess protein abundance of four sarcomeric proteins using commercially available antibodies (CAPZA1 (#ab125972), MYOT (#ab68915), MYOZ1 (#ab103767), PDLIM3 (#ab83259) all from Abcam). Unfortunately, the results were difficult to interpret and quantify, in particular due to multiple bands recognized in total muscle lysates. Limited amount of material prevented us to repeat this part of the study with antibodies from other sources. Therefore, we focused our analysis on mRNA expression in skeletal muscle of NGT individuals with the three different R577X genotypes to determine the relationship between the ACTN3 R577X polymorphism and sarcomeric protein mRNA.

In conclusion, the prevalence of the ACTN3 577XX genotype is higher in T2D patients, but the genotype distribution is unrelated to metabolic control or obesity. Abundance of mitochondrial proteins NDUFB8 and COX2 was reduced in skeletal muscle from T2D patients carrying the X allele. *α*-actinin 3 deficiency is associated with increased mRNA expression of sarcomeric proteins in human muscle. These transcriptional changes in structural proteins associated with lack of *α*-actinin 3 may provide a molecular basis for differences in exercise performance and skeletal muscle function in carriers of the ACTN3 R577X polymorphism.
